# Activity-Based Tracking of Glycan Turnover in Microbiomes

**DOI:** 10.1021/jacs.5c07546

**Published:** 2025-07-08

**Authors:** Conor J. Crawford, Greta Reintjes, Vipul Solanki, Manuel G. Ricardo, Jens Harder, Rudolf Amann, Jan-Hendrik Hehemann, Peter H. Seeberger

**Affiliations:** † 28321 Max Planck Institute for Colloids and Interfaces, Potsdam 14476, Germany; ‡ Faculty of Biology/Chemistry, 9168 University of Bremen, Bremen 28359, Germany; § 28267 Max Planck Institute for Marine Microbiology, Bremen 28359, Germany; ∥ MARUM,Center for Marine Environmental Sciences, University of Bremen, Bremen 28359, Germany; ⊥ Institute for Chemistry and Biochemistry, Freie Universität Berlin, Berlin 14195, Germany

## Abstract

Glycans shape microbiomes in the ocean and the gut, driving key steps in the global carbon cycle and human health. Yet, our ability to track microbial glycan turnover across microbiomes is limited, as identifying active degraders without prior genomic knowledge remains a key challenge. Here, we introduce an activity-based fluorescence resonance energy transfer (FRET) probe that enables direct visualization and quantification of glycan metabolism in complex microbial communities. As a proof of concept, we investigated α-mannan degradation, a prominent polysaccharide in algal blooms. Using automated glycan assembly, we synthesized a mannan hexasaccharide bearing a fluorescein–rhodamine FRET pair. The probe was validated using a recombinantly expressed *endo*-α-mannanase (GH76) from sp. Hel_I_6. It was shown to function in cell lysates, pure cultures, and complex microbiomes (via plate assays and microscopy). This probe enabled spatiotemporal visualization of in situ α-mannan turnover in a marine microbiome. Glycan FRET probes are versatile tools for tracking glycan degradation across biological scales from single enzymes to microbiomes.

## Graphical abstract

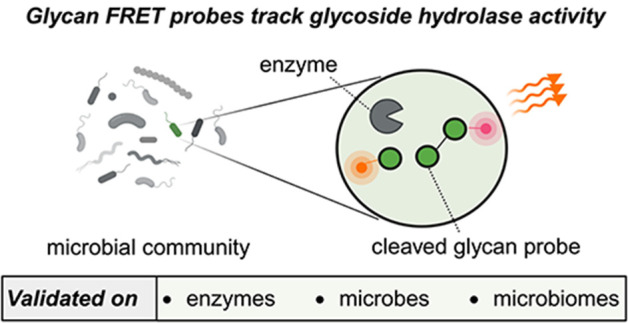

## Introduction

Microbes are central to environmental and human health,^1,2^ and glycan-derived carbon is a key factor shaping microbial community composition in ecosystems from the ocean to the human gut.^3–5^ Algae invest up to 80% of their fixed organic carbon into glycans,^6^ and during algal blooms, microbes compete for access to specific glycan structures in this complex chemical matrix. Studying these dynamics typically relies on metagenomics, metatranscriptomics and the axenic cultivation of microbes. However, sequence-based methods are biased toward known sequences,^7,8^ and many microbes cannot be cultured by standard techniques.^9,10^ In addition, our understanding of nature’s enzymatic diversity for glycan degradation is incomplete.^11^ Marine ecosystems represent fertile ground for discovery due to their structurally complex polysaccharides (e.g., fucoidan) and novel carbohydrate-active enzymes with implications for carbon cycling and climate modeling.

Chemical probes offer a strategy for directly detecting enzymatic activity at the cellular level, independent of genomic or cultivation-based information.^12–16^ Fluorescently labeled polysaccharides (FLAPS)^17^ and related oligosaccharide probes^18,19^ allow single-cell visualization of carbohydrate metabolism in complex communities. However, these probes rely on heterogeneous biologically extracted glycans that are fluorescently labeled by nonsite-selective chemical conjugation (e.g., cyanogen bromide), which can challenge reproducibility and precise interpretation. Furthermore, these tools detect signal accumulation rather than direct catalytic activity, yet they have enabled the study of microbial glycan metabolism in diverse ecosystems, including the rumen,^20^ the ocean,^17,21^ and the human gut.^18,22^

Here, we report an activity-based glycan probe strategy based on fluorescence resonance energy transfer (FRET). Using automated glycan assembly (AGA), we synthesized a bifunctional mannan oligosaccharide bearing a donor–acceptor fluorophore pair. This probe selectively detects *endo*-acting enzymes, which initiate the breakdown of complex polysaccharides into oligosaccharides for transport and metabolism. We investigated α-mannan turnover due to its dual role in host-associated and environmental microbiomes, with fungal α-mannan acting as a prebiotic in the human gut^23,24^ and metagenomic analyses identifying α-mannan turnover in algae blooms.^25^ This chemically defined FRET probe enabled the functional interrogation of mannan degradation across three biological scales: purified enzymes, marine bacterial strains, and a marine microbial community.

## Results and Discussion

### Structure-Guided Design of a FRET-Active α-Mannan Probe

Turnover of α-mannan polysaccharides has been studied using metagenomic datasets from spring phytoplankton blooms in the North German Sea.^25^ Among the glycan-degrading taxa, sp. Hel_I_6 encodes an *endo*-α-1,6-mannanase (GH76) sharing 27% sequence identity with homologues from the human gut microbiota (Figure 1a). Guided by the crystal structure of the marine GH76 enzyme in complex with mannotetraose,^25^ we designed a mannan-based FRET probe to match the active site topology and enable fluorophore placement (Figure 1b, c).

**Figure 1:**
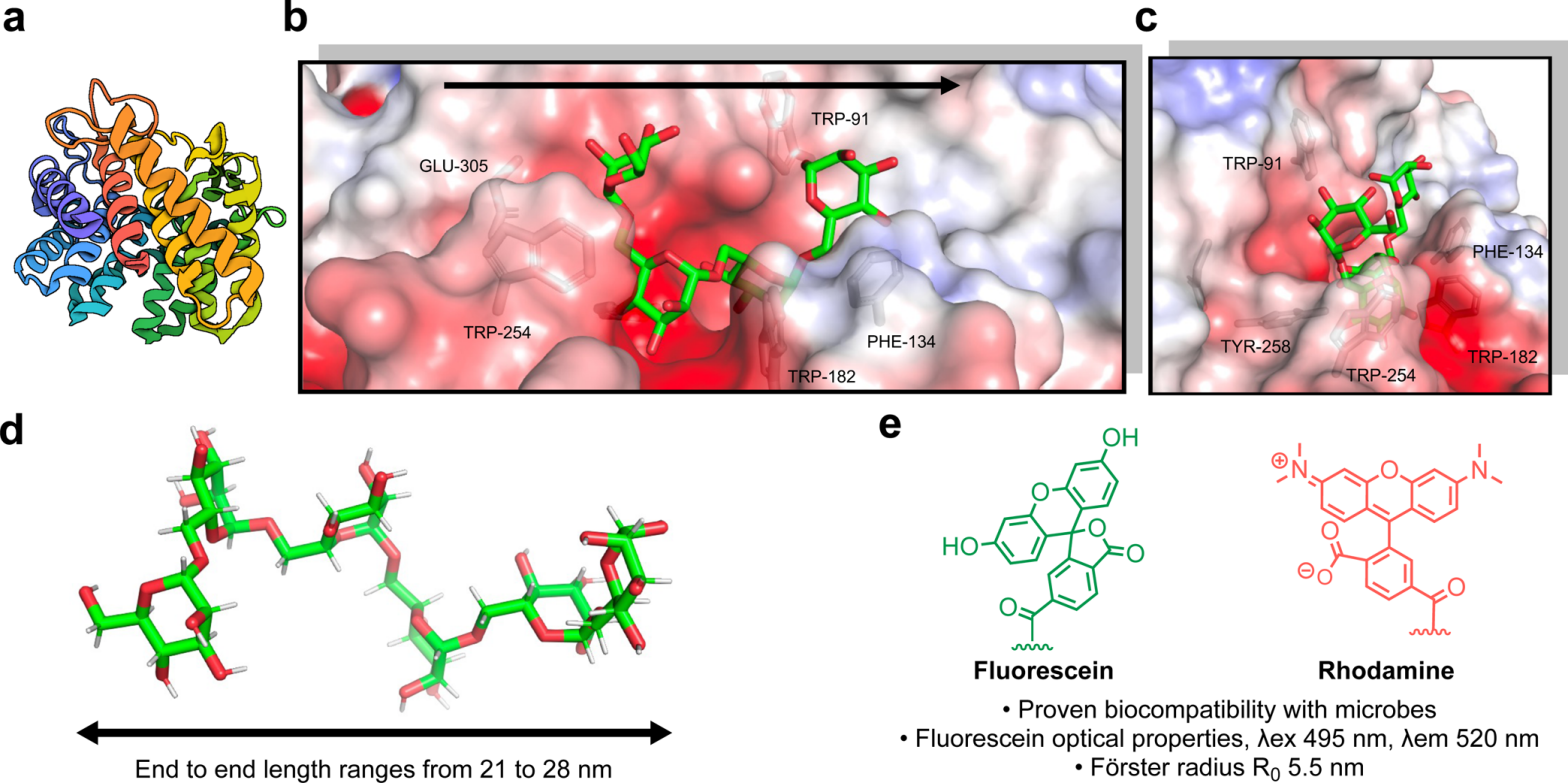
Structure-guided design of a FRET mannan probe. (a) GH76 from sp. Hel_I_6 (PDB ID: 6SHD)^25^ represented using rainbow protein structure. (b) Solved crystal structure of GH76 in complex with a mannan tetrasaccharide, aromatic side chains, and arrow provide orientation. (c) Alternative view of the active site, highlighting the tunnel-like topology and solvent-exposed region that provide good exit vectors for the fluorophore and quencher. (d) Conformational diversity of 1,6-linked mannans, ranging from 21 to 28 nm in end-to-end length. (e) Fluorescein–rhodamine FRET pair has proven biocompatibility in microbial communities, good optical properties, and function within the length of the mannan hexasaccharide.

The enzyme’s partially solvent-exposed, tunnel-like active site suggested that the installation of a fluorophore–quencher pair via flexible linkers would be compatible with enzyme recognition. To optimize both substrate binding and FRET efficiency, we selected a hexasaccharide scaffold, long enough to span the active site while positioning the fluorophores within the Förster radius (*R*_0_ ≈ 5.5 nm) (Figure 1d).^26,27^ Given the structural flexibility of α-1,6-mannans, this length was predicted to balance recognition and quenching efficiency. We chose fluorescein and tetramethylrhodamine as the FRET pair due to their spectral compatibility and reported biocompatibility in marine bacterial systems (Figure 1e). The quencher on the nonreducing end blocks *exo*-glycosidase activity and ensures cleavage of the internal glycosidic bonds, favoring detection of *endo*-acting enzymes such as GH76, which are typically located in the periplasm or at the cell surface depending on species-specific transport and localization mechanisms.^28,29^

Site-selective labeling at the reducing and nonreducing termini required the installation of two orthogonal functional handles. As oligosaccharides lack inherent termini like those in peptides, we installed a 6-amino-6-deoxy monosaccharide at the nonreducing end and a 5-aminopentanol-modified polystyrene resin at the reducing end during automated glycan assembly (AGA).^30,31^ A Boc-protected amine was selected for the nonreducing terminus, allowing for selective deprotection and sequential fluorophore conjugation following global deprotection and benzyloxycarbonyl (CBz) cleavage.

One practical challenge was the benzyl ether protecting groups commonly used during AGA.^32^ Cleavage of these ethers requires palladium-catalyzed hydrogenolysis, a step that is incompatible with sensitive aromatic fluorophores. To preserve dye integrity, all conjugation steps were deferred until the final stages of the synthesis.

### Automated Synthesis of a Fluorescence-Quenched Mannan Probe

The bifunctional α-mannan hexasaccharide probe was synthesized using a custom-built automated glycan assembly (AGA) synthesizer.^31^ The polystyrene solid support contained a photocleavable 5-aminopentanol linker, and the glycosylations were carried out over six coupling cycles.

In the first five cycles, an acidic wash with trimethylsilyl trifluoromethanesulfonate (TMSOTf) was followed by *N*-iodosuccinimide (NIS) and triflic acid (TfOH) promoted glycosylation with building block **1** (T_1_ −20 °C for 15 min, T_2_ 0 °C for 35 min) (CH_2_Cl_2_:Dioxane, 2:1) (Figure 2a,b). The final coupling with building block **2** was performed under identical conditions. After each glycosylation, unreacted nucleophiles were capped using acetic anhydride and methanesulfonic acid to ensure minimal elongation of deletion sequences.^33^ The temporary Fmoc carbonate (OFmoc) protective groups were then removed by using a 20% piperidine solution in dimethylformamide, exposing the acceptor for the subsequent coupling. The automated synthesis proceeded with high purity and minimal deletion sequences (Figure 2c and Figures S1 and S2).

**Figure 2:**
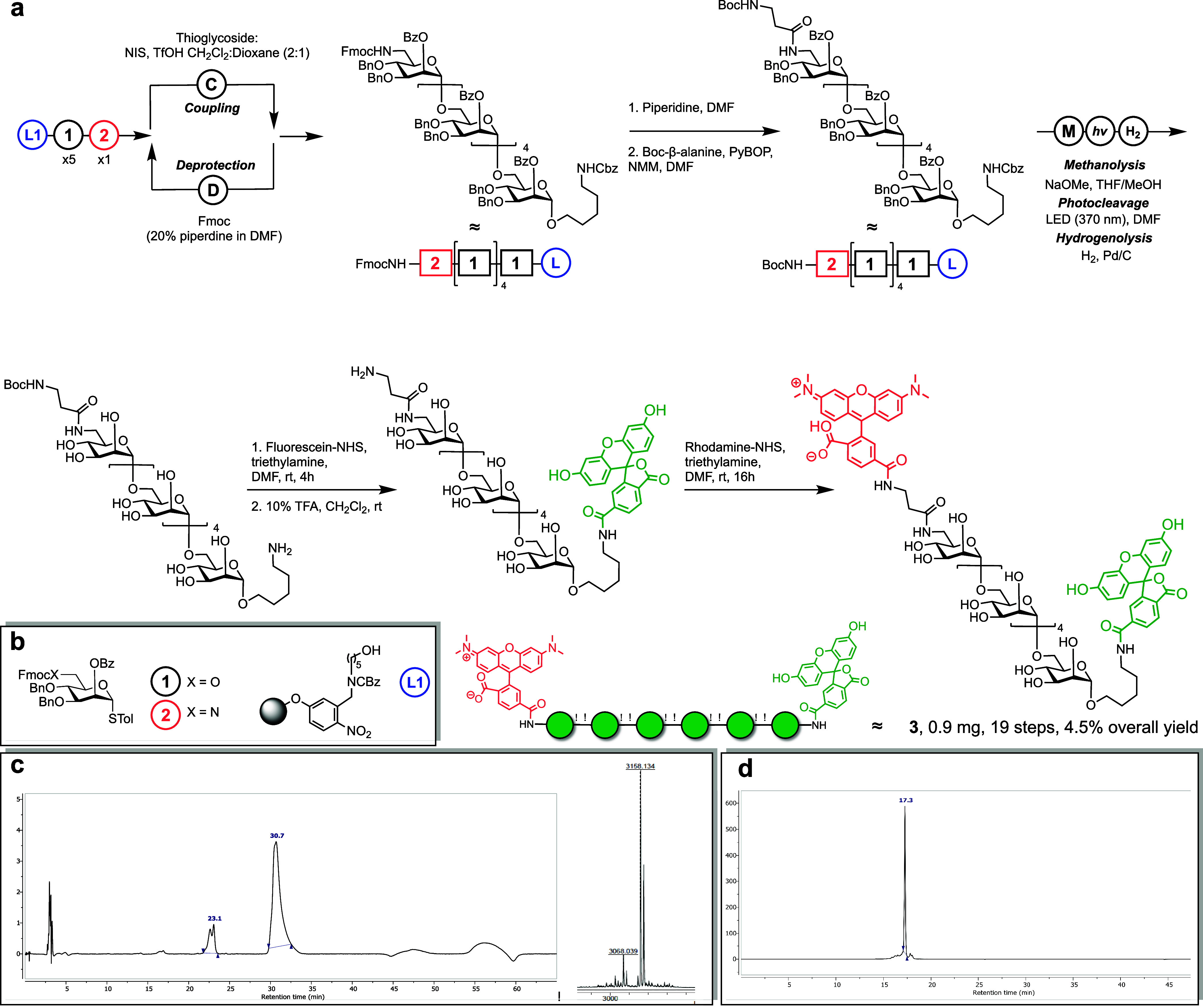
Automated glycan assembly of the fluorescence quenched mannan probe. (a) Automated assembly of a fluorescent quenched probe. (b) Building blocks and resin were required for synthesis. (c) Left: crude ELSD trace of hexasaccharide after automated glycan assembly (SI Method 1, YMC-Diol-300). Right: MALDI-TOF of hexasaccharide, calculated C_190_H_186_N_2_NaO_40_ [M + Na]^+^ 3158.2480 *m*/*z*, found 3158.134 *m*/*z*. For full spectrum, see Figure S2. (d) UV-trace (566 nm) of purified **3** on an HPLC (SI Method 2, Luna C5 column).

At key stages, UV-based microcleavage analysis was employed to monitor synthesis progress by HPLC and mass spectrometry (Figures S3–S5). To enable selective late-stage conjugation of the rhodamine quencher, the nonreducing end Fmoc-amine was cleaved using 20% piperidine in DMF and functionalized with a Boc-protected β-alanine spacer via PyBOP-promoted peptide coupling. Base-sensitive esters were cleaved by on-resin methanolysis (Figure S3), and the hexasaccharide was released from the solid support using an LED lamp (380 nm).^34^ Benzyl ether protecting groups were then removed by palladium-catalyzed hydrogenolysis.^35,36^

Following the successful deprotection of the bifunctional hexasaccharide (Figure S4), the conjugation of fluorescent dyes proceeded in a stepwise fashion. First, fluorescein-NHS ester was reacted with the reducing-end amine (Figure S5). After purification (SI Method 3, Luna C5 column), the Boc group on the nonreducing end was cleaved under acidic conditions to expose the second amine for conjugation with the rhodamine quencher. Final purification by HPLC (SI Method 3, Luna C5 column) yielded fluorescence-quenched mannan probe **3** (0.9 mg, 19 steps, 4.5% overall yield).

### Fluorescence-Quenched Mannan Probe Detects Glycoside Hydrolase Activity

The fluorescence-quenched α-mannan probe exhibited a quenching efficiency of 68.9%, representing an approximate 3-fold reduction in emission intensity compared to free fluorescein (Figure 3a). This quenching efficiency proved sufficient for our experiments, particularly given the intrinsic flexibility of α-1,6-linked mannans.

**Figure 3:**
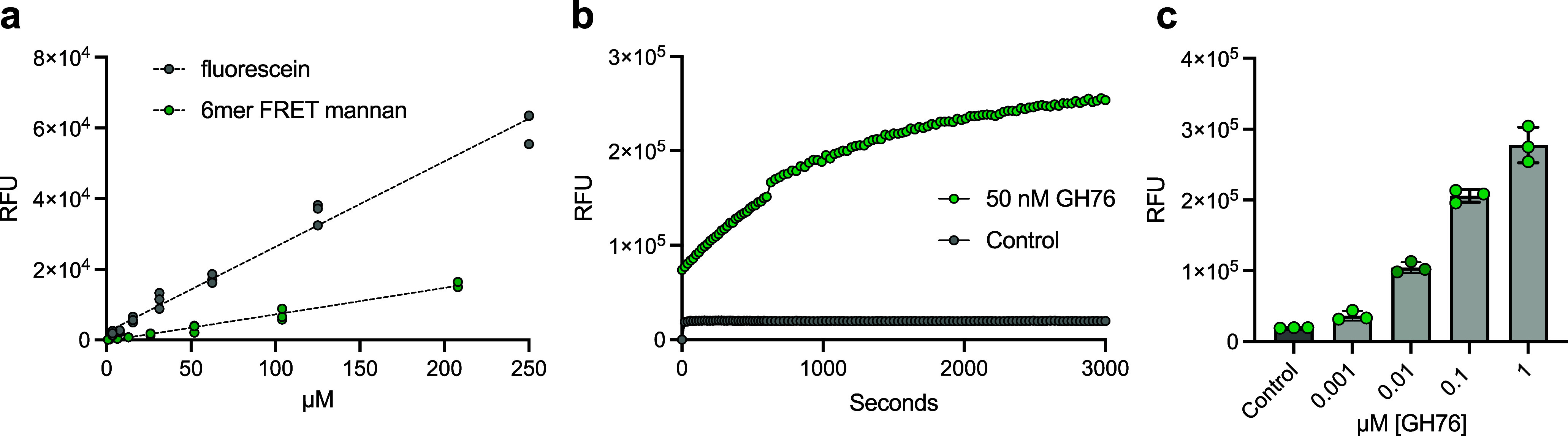
Biochemical characterization of GH76 from sp. Hel_I_6. (a) Quenching efficiency of the fluorescence-quenched mannan probe (68.9%) quantified by comparing the slopes of linear regression of fluorescein and probe emission intensities. Dotted line depicts linear regression analysis with individual data points shown. (b) Incubation of 10 nM FRET mannan probe with 50 nM GH76 results in a time-dependent fluorescence increase. Fluorescence at 520 nm indicates glycan hydrolysis (Excitation 495 nm). (c) Increasing concentration of GH76 results in increased fluorescence intensity when incubated with 10 nM probe at 500 s. Shown is the mean, and error bars represent standard deviation from the mean (*n* = 3).

When incubated with recombinant GH76, the probe showed fluorescence recovery in both time- and concentration-dependent manners (Figure 3b). Enzymatic cleavage was detectable at probe concentrations as low as 1 nM, making the probe suitable for detecting GH76 activity in environmental samples (Figure 3c). Furthermore, increasing the concentration of GH76 resulted in increased fluorescence intensity (Figure 3c). Kinetic analysis via nonlinear regression (*R* ^2^ = 0.97) revealed a catalytic efficiency of 0.663 μM^–1^ s^–1^ for GH76 cleavage (Figure S6). Notably, incubation with the β-1,3-*endo*-laminarinase FbGH17a did not lead to probe cleavage, confirming the specificity of the probe for GH76.^37^

### Activity-Based Sensing of α-Mannan Degradation in Complex Proteomes

The activity-based nature of the probe in complex proteomes was tested by exposing it to cell lysates from four marine bacteria species that have fully sequenced genomes and annotated polysaccharide utilization loci (PUL): sp. Hel_I_6, , , and *Hel1_33_131*. Cells were harvested in mid log phase, centrifuged, lysed with BugBuster, and incubated with 1 μM probe in phosphate-buffered saline buffer (50 mM PBS, pH 7.4) for 1 h at 20 °C prior to plate reader fluorescence analysis (λ_ex_ 495 nm, λ_em_ 520 nm). PUL analysis predicted that only sp. Hel_I_6 encodes a GH76 enzyme capable of degrading α-mannan.^38^ To induce the expression of carbohydrate-active enzymes, all strains were grown in low-carbon medium (HaHa3 V) with 100 mg/L yeast α-mannan. Consistent with these predictions, sp. Hel_I_6 lysates showed a significant 7-fold fluorescence increase after incubation with the FRET probe, while lysates from the other three bacteria did not show a significant fluorescence increase (Figure 4a). Heat-inactivated controls confirmed that the fluorescence increase was enzyme-dependent.

**Figure 4:**
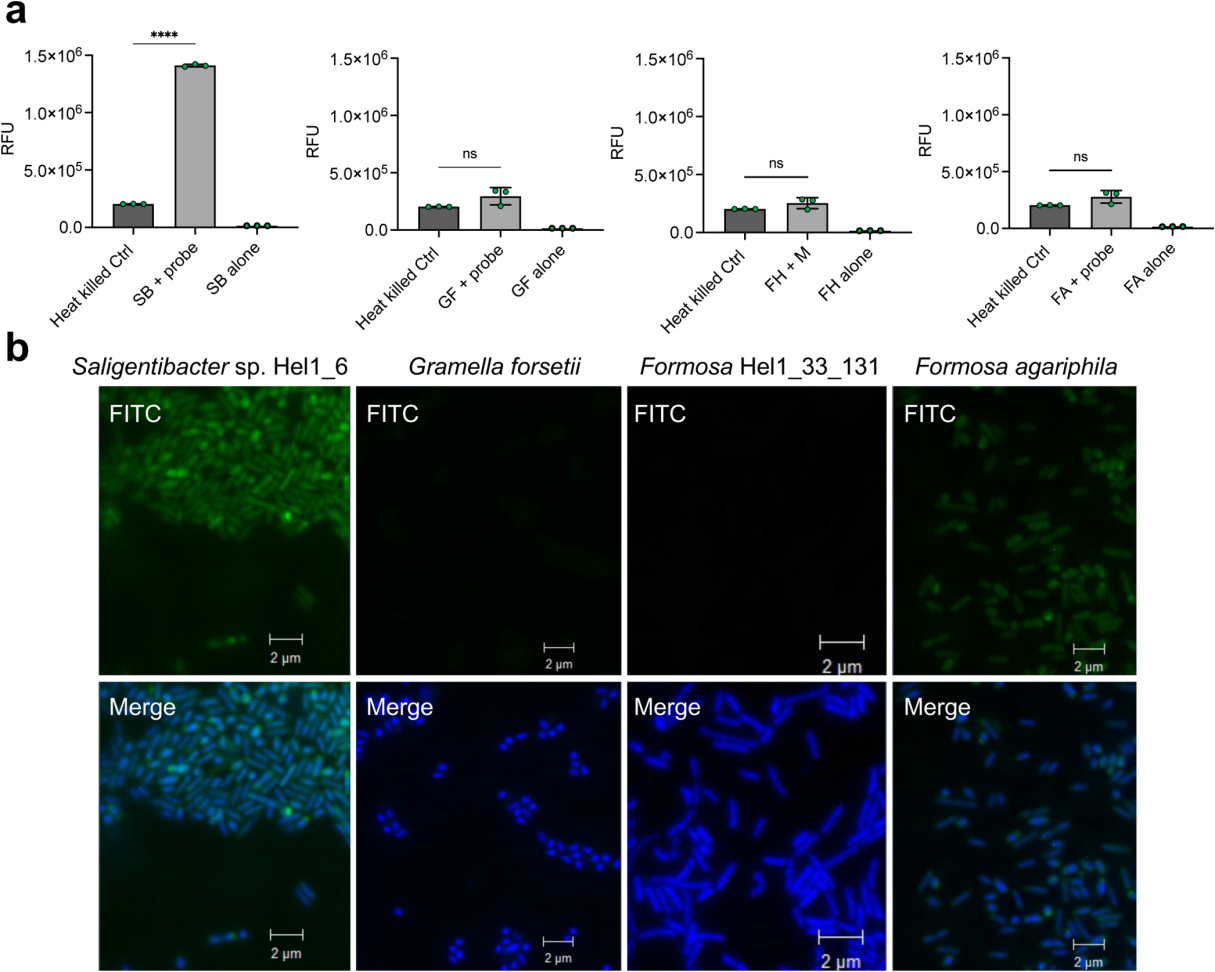
Detection of mannan degradation in marine bacterial lysates and cultures using FRET probes. (a) Fluorescence signal following incubation of mannan probe (1 μM) with cell lysates from sp. Hel_I_6 (SB), (GF), *Hel1_33_131* (FH), and (FA). The probes were excited at 495 nm, and emission was detected at 520 nm. Cell lysates were prepared using BugBuster reagent. A negative control (Ctrl) of heat-killed cells (95 °C for 30 min) of each strain were incubated with 1 μM mannan probe. The background fluorescence of each strain was recorded and annotated as strain acronym alone (e.g., SB alone). Experiments were performed as independent triplicates (*n* = 3) and error bars represent the standard deviation of the mean. (b) Representative microscopy of four species of marine bacteria. Green (FITC) corresponds to mannan probe signal, and blue (DAPI) labels the cell nuclei. Merge is the overlay of FITC and DAPI signals. Scale bar: 2 μM.

Microscopy of the coccoid and rod-shaped .*Hel1_33_131* cells revealed no fluorescence from the probe, confirming the absence of active glycoside hydrolase enzymes, as predicted.^38,39^ Cells were incubated with 2.5 μM FRET probe in PBS (50 mM, pH 7.4) for 60 min, washed, fixed with formaldehyde, and imaged under identical settings. In sp. Hel_I_6 fluorescence was detected in the periplasmic space and is consistent with the cellular localization of the GH76 *endo*-α-mannanase (Figure 4b).^17^ Super-resolution STED suggested discrete periplasmic foci, though photobleaching limited image quality (Figure S7).^22^ Future probe could use more photostable dyes to improve imaging.^40–42^ Although periplasmic localization is consistent with uptake and internal hydrolysis by an *endo*-acting enzyme, we cannot exclude the possibility of initial extracellular cleavage followed by uptake of labeled fragments. The observed signal may reflect a combination of both processes.

Periplasmic fluorescence in the FITC channel for suggested GH76-like activity, although the signal was weaker and more sporadic than in sp. Hel_I_6, occurring in only 212 of 4,470 cells (≈4.7%) (Figure 4b andFigure S8). Follow-up BLASTP and dbCAN genome searches did not identify high-homology GH76 hits. The absence of a high-sequence homology GH76 enzyme aligns with recent findings that carbohydrate-active enzymes have more diverse catalytic mechanisms and consequently more diverse sequences.^11^ may metabolize an unidentified marine mannan similar to the recently discovered sulfated mannan from .^43^ We note that this probe is designed for *endo*-selective glycosidase sensing, but its uptake may require specific transporters, and differences in signal intensity across species may reflect variable expression or the absence of such systems.

### Spatiotemporal Resolution of Glycan Turnover in a Marine Microbiome

Next, we assessed the probe activity in an environmental seawater microbiome. Cells were cultured under either α-mannan (specific stimulus) or yeast extract (general carbon stimulus) for 3 days at 20 °C (Figure 5a) and then exposed to the α-mannan FRET probe (5 μM). Samples were taken at 15 min, 30 min, 1.5 h, 3 h, and 7 h and analyzed by automated microscopy.

**Figure 5:**
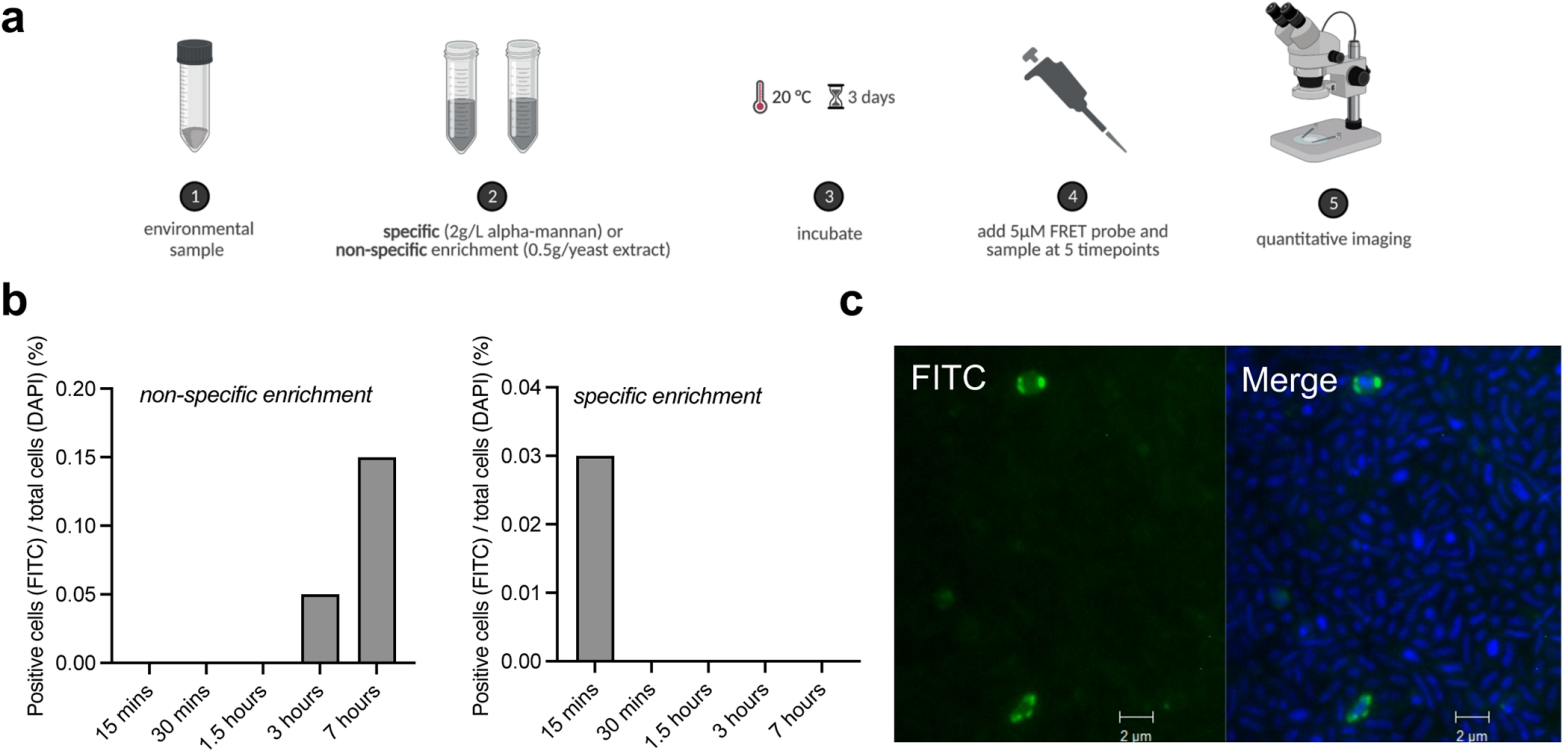
Visualization of α-mannan utilization in a complex microbial community. (a) Workflow for sensing α-mannan carbon in microbial communities. (b) Quantification of nonspecific (0.5 g/L yeast extract) and specific (2 g/L α-mannan) culture conditions on the hydrolysis of the mannan probe in a marine microbial community. (c) Shown is a representative microscopy image of a selective hydrolysis of the mannan probe in a marine microbiome from the nonspecific enrichment sample at 7 h. Green (FITC) indicates mannan probe hydrolysis, and blue (DAPI) labels cell nuclei. Merge is overlay of FITC and DAPI signals. Constant exposure times were used, and fluorescence signal thresholds were determined from control cells not exposed to FRET probe. Experiment is representative of duplicate. Scale bar: 2 μm.

The turnover activity of α-mannan was dependent on prior exposure to the α-mannan stimulus (Figure 5b). In unprimed marine communities, degradation occurred gradually, peaking at 7 h, with 5.91 × 10^4^ (0.15%) cells displaying α-mannan activity (Table S1). In contrast, α-mannan-stimulated communities showed complete turnover within 15 min. This more rapid metabolic response is likely due to the preinduction of glycoside hydrolases and transporters, resulting in accelerated metabolism of the mannan probe. A more gradual and heterogeneous uptake occurs in communities grown on the general carbon stimulus (yeast extract), which contains diverse glycans including α-mannans. The absence of fluorescent cells in the α-mannan-stimulated community after 15 min may reflect a shift in glycan acquisition strategies, from internal (aka. selfish uptake) to extracellular degradation. In this scenario, the fluorescent mannan would not be associated and would prevent visualization. Alternatively, the community has completed the turnover of the probe in less than 30 min, and the released dyes have been exported from the periplasm, leaving the cells apparently inactive.

Fluorescent cells were predominantly coccoid in shape, with a polar fluorescence pattern indicative of periplasmic localization (Figure 5c).^22^ Overall, these results show that the probe can sense carbon source-driven shifts in glycan turnover, enabling spatiotemporal and activity-based resolution of carbohydrate metabolism in microbiomes.

## Conclusion

A mannan oligosaccharide fluorescence resonance energy transfer (FRET) probe was prepared by automated glycan assembly. This probe enabled the quantitative analysis of *endo*-acting glycoside hydrolase (GH76) activity, the functional investigation of polysaccharide utilization loci (PUL) predictions in four marine bacterial species, and the spatiotemporal visualization of α-mannan turnover in marine microbial communities. This proof-of-concept study highlights the utility of FRET-based glycan sensors for detecting and quantifying glycan degradation across biological scales, from enzymes to complex microbiomes. We expect that expanding and optimizing this platform to other glycan structures will uncover new microbial metabolic networks with applications in environmental monitoring, microbiome engineering, and microbial ecology.

## Supplementary Material



## Notes

EGeneral materials and methods; resin and building blocks; automated glycan assembly; preparation of reagent solutions; solid-phase synthesis; solution-phase synthesis; HPLC analysis and purification; compound characterization; overexpression and purification of GH76; bacteria culture; FRET mannan uptake studies with marine bacteria; epifluorescence microscopy; microbial community culture; automated fluorescence microscopy; super resolution STED microscopy (PDF)
