# Supplementary material for: Activity-Based Tracking of Glycan Turnover in Microbiomes
Source: J Am Chem Soc. 2025 Jul 8;147(29):25799–805. doi: 10.1021/jacs.5c07546 (PMC12291463; doi:10.1021/jacs.5c07546)
Supplement: Supplementary file 1 [file ja5c07546_si_001.pdf]

Supporting Information

Activity-based tracking of glycan turnover in microbiomes

Conor J. Crawford,[1] Greta Reintjes,[2] Vipul Solanki,[3,4] Manuel G. Ricardo,[1] Jens Harder,[3] Rudolf Amann,[3] Jan-Hendrik Hehemann,[2,3,4] Peter H. Seeberger[1,5]*

[1]Max Planck Institute for Colloids and Interfaces, Potsdam, 14476, Germany. [2]Faculty of Biology/Chemistry, University of Bremen, Bremen, 28359, Germany. [3]Max Planck Institute for Marine Microbiology, Bremen, 28359, Germany. [4]MARUM, Center for Marine Environmental Sciences, University of Bremen, Bremen, 28359, Germany. [5]Institute for Chemistry and Biochemistry, Freie Universität Berlin, Berlin, 14195, Germany.

Table of Contents

General materials and methods 3

Resin and building blocks 4

SI Figures 5

Automated glycan assembly 14

Preparation of reagent solutions 14

Solid-phase synthesis 18

Solution-phase synthesis 19

HPLC analysis and purification 19

Compound characterization 20

Overexpression and purification of GH76 25

Bacteria culture 26

FRET mannan uptake studies with marine bacteria 26

Epifluorescence microscopy 27

Microbial community culture 27

Automated fluorescence microscopy 27

Super resolution STED microscopy 28

References 29

General materials and methods

All chemicals used were reagent grade and used as supplied unless otherwise noted. The automated syntheses were performed on a home-built synthesizer developed at the Max Planck Institute of Colloids and Interfaces. Analytical thin-layer chromatography (TLC) was performed on Merck silica gel 60 F254 plates (0.25 mm). Compounds were visualized by UV irradiation or dipping the plate in a 5% H_2SO_4 ethanol solution. Flash column chromatography was carried out on automated Grace flash chromatography system. Analysis and purification by normal and reverse phase HPLC was performed by using an Agilent 1200 series. Products were lyophilized using a Christ Alpha 2-4 LD plus freeze dryer. ^{1}H, ^{13}C and HSQC NMR spectra were recorded on a Varian 400MR (400 MHz), Varian 600MR (600 MHz), or Bruker Biospin AVANCE700 (700 MHz) spectrometer. Signals are reported in terms of chemical shift [δ in parts per million (ppm)] relative to tetramethylsilane (TMS) or in D_2O using the solvent as the internal standard in ^{1}H NMR (D2O: 4.79 ppm ^{1}H). NMR data is presented as follows: Chemical shift, multiplicity (s = singlet, d = doublet, t = triplet, dd = doublet of doublet, m = multiplet and/or multiple resonances), coupling constant in Hertz (Hz), integration. All NMR signals were assigned on the basis of ^{1}H NMR, ^{13}C NMR, COSY, TOCSY and HSQC experiments. ‡Carbon assignments were extracted from HSQC experiment. High resolution mass spectra were obtained using a 6210 ESI-TOF mass spectrometer (Agilent) and a MALDI-TOF autoflexTM (Bruker). MALDI and ESI mass spectra were run on IonSpec Ultima instruments. The matrix used for MALDI-MS was 2,5-dihydroxy-benzoic acid (DHB).

Resin and building blocks

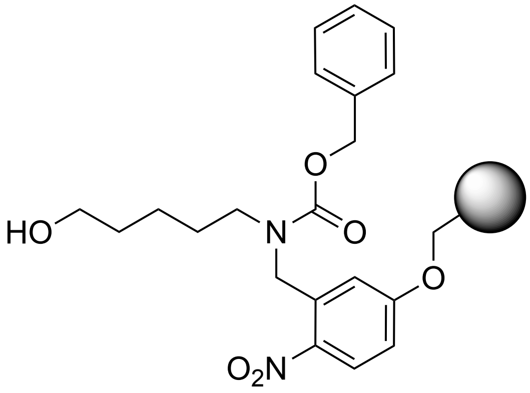

1

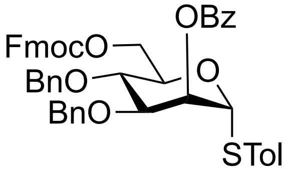

2

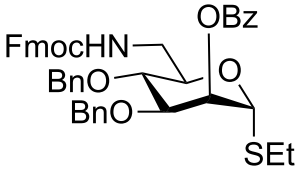

Building blocks and resins were prepared using published procedures, or purchased from GlycoUniverse GmbH & CO KGaA (Germany).[1,2]

SI Figures

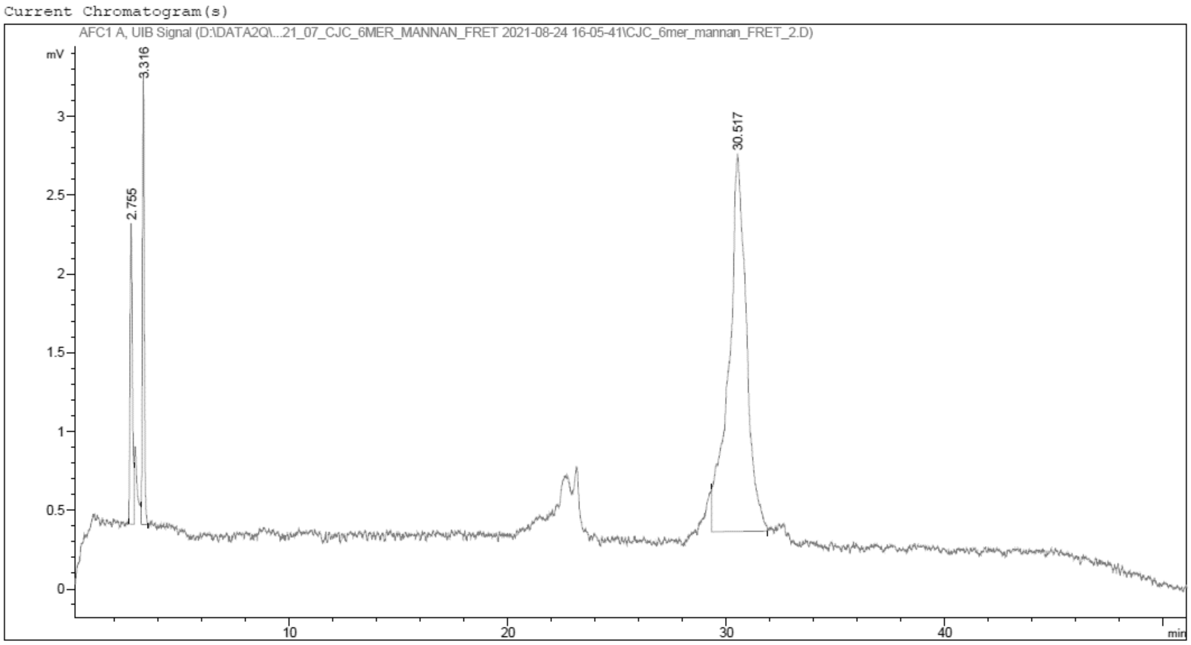

SI Figure 1. HPLC trace of crude automated glycan assembly of *hetero-bifunctional* mannan oligosaccharide.

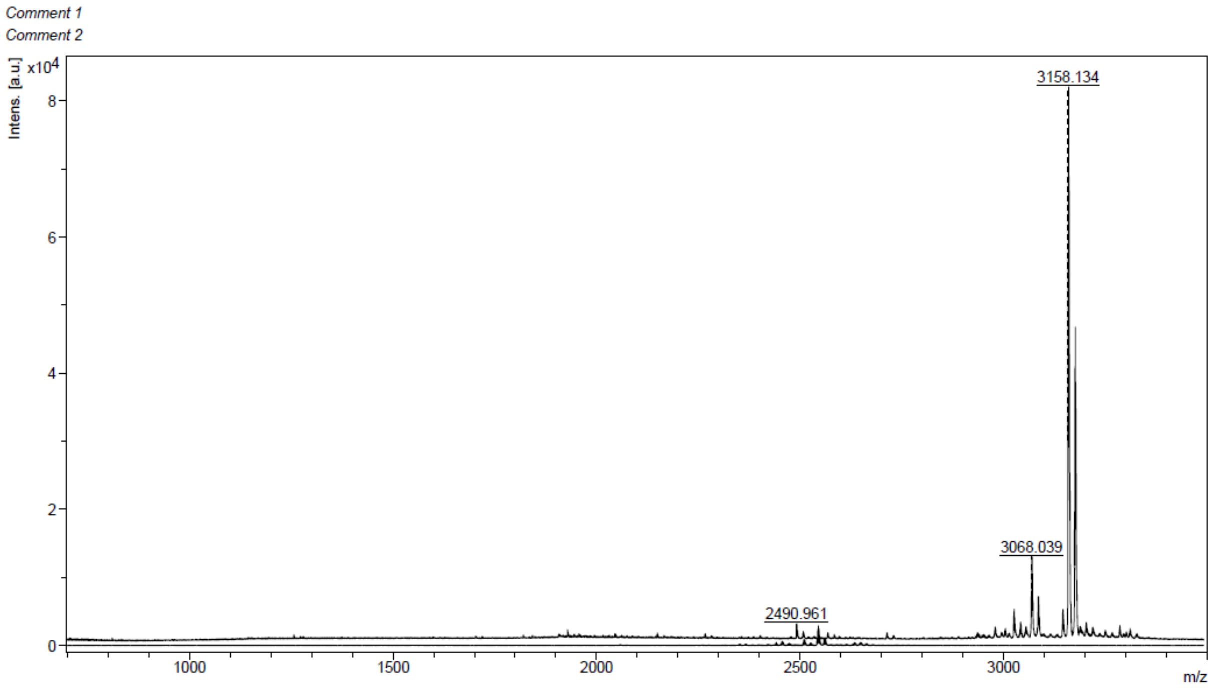

SI Figure 2. MALDI-TOF of crude automated glycan assembly of *hetero-bifunctional* mannan oligosaccharide. Expected $[M+Na]^+$ 3158.248, observed 3158.450.

a

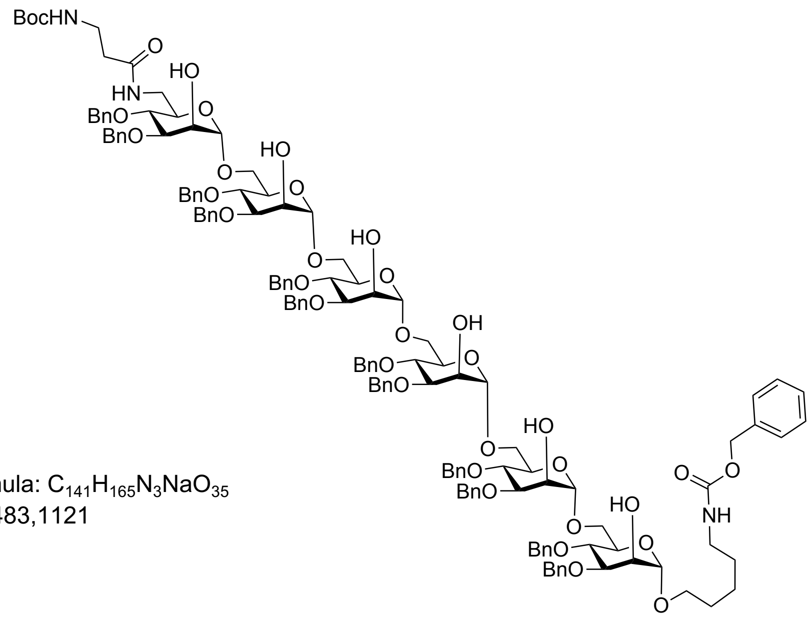

Chemical Formula: $C_{141}H_{165}N_3NaO_{35}$
Exact Mass: 2483,1121

b

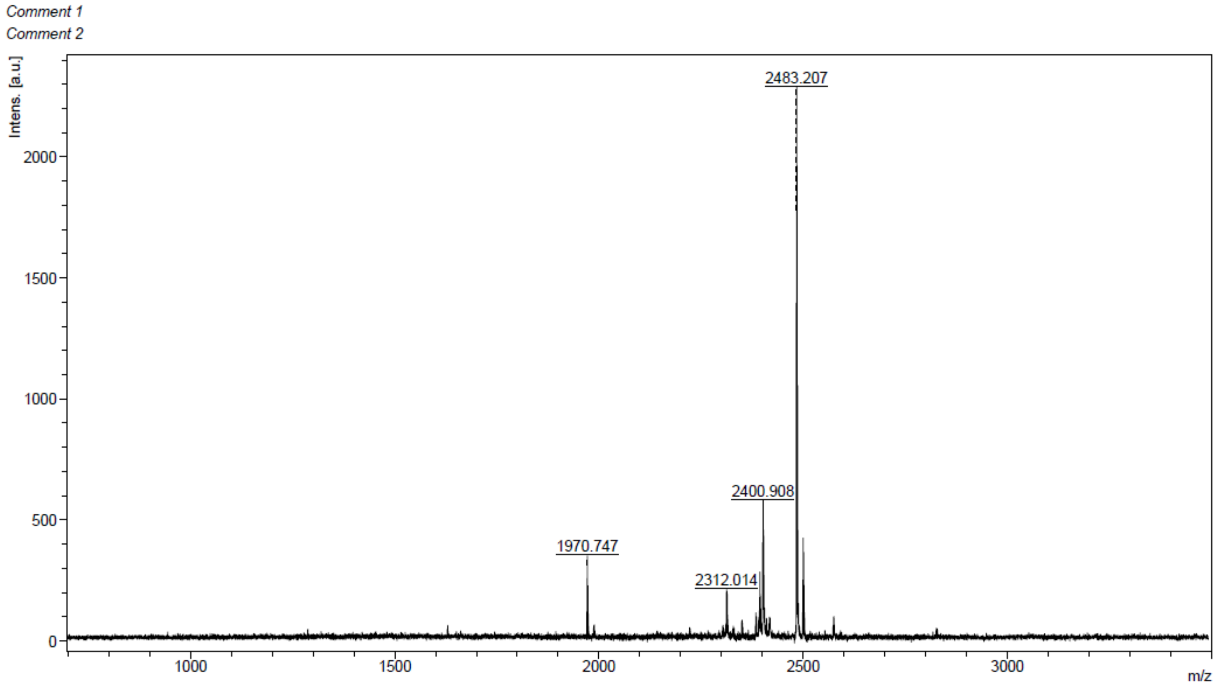

SI Figure 3. Microcleavage analysis after methanolysis. a Chemical structure of Mannan oligosaccharide after on resin methanolysis. **b** MALDI of oligosaccharide *m/z* = 2483 $[M+Na]^{+}$.

a

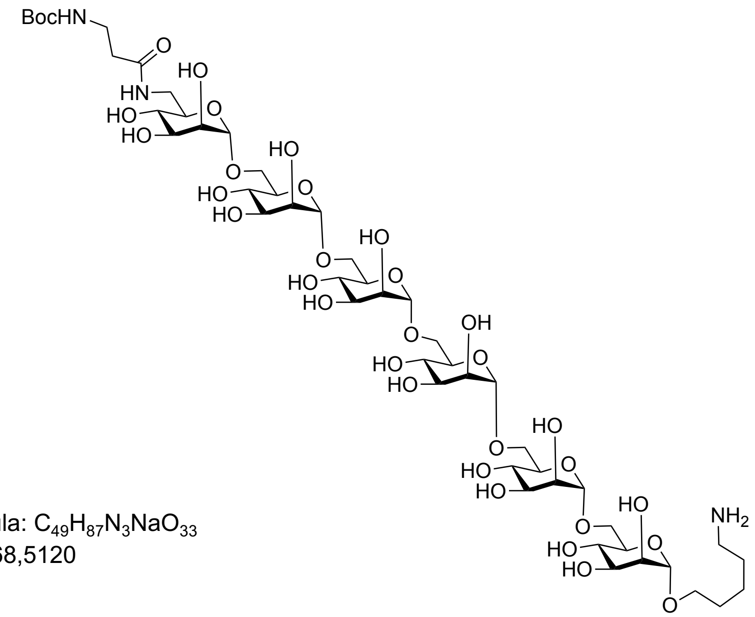

Chemical Formula: $C_{49}H_{87}N_3NaO_{33}$
Exact Mass: 1268,5120

b

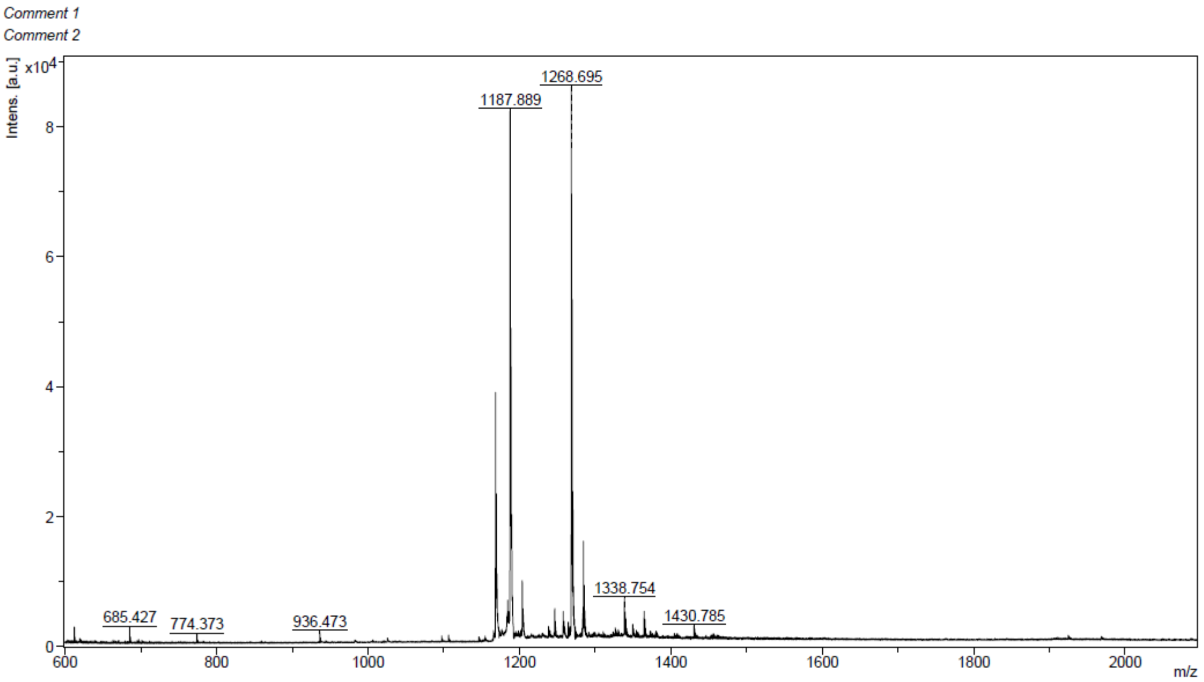

SI Figure 4. Microcleavage analysis after methanolysis. a Chemical structure of Mannan oligosaccharide after hydrogenolysis. **b** MALDI of oligosaccharide *m/z* = 1268 $[M+Na]^+$. Note during ionisation and or sample preparation acid labile Boc group was cleaved, 1187 *m/z*.

a

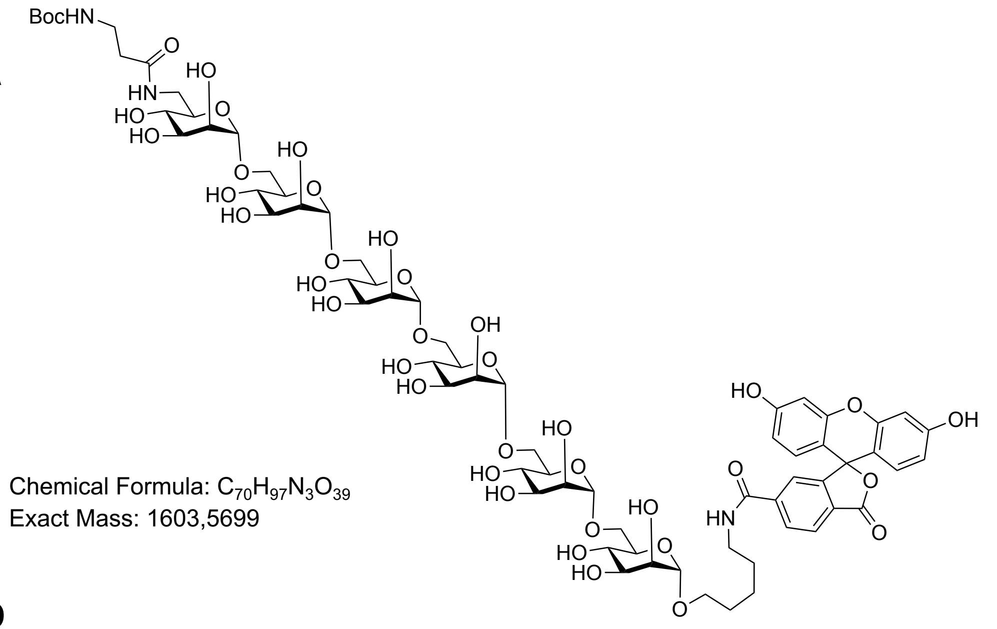

b

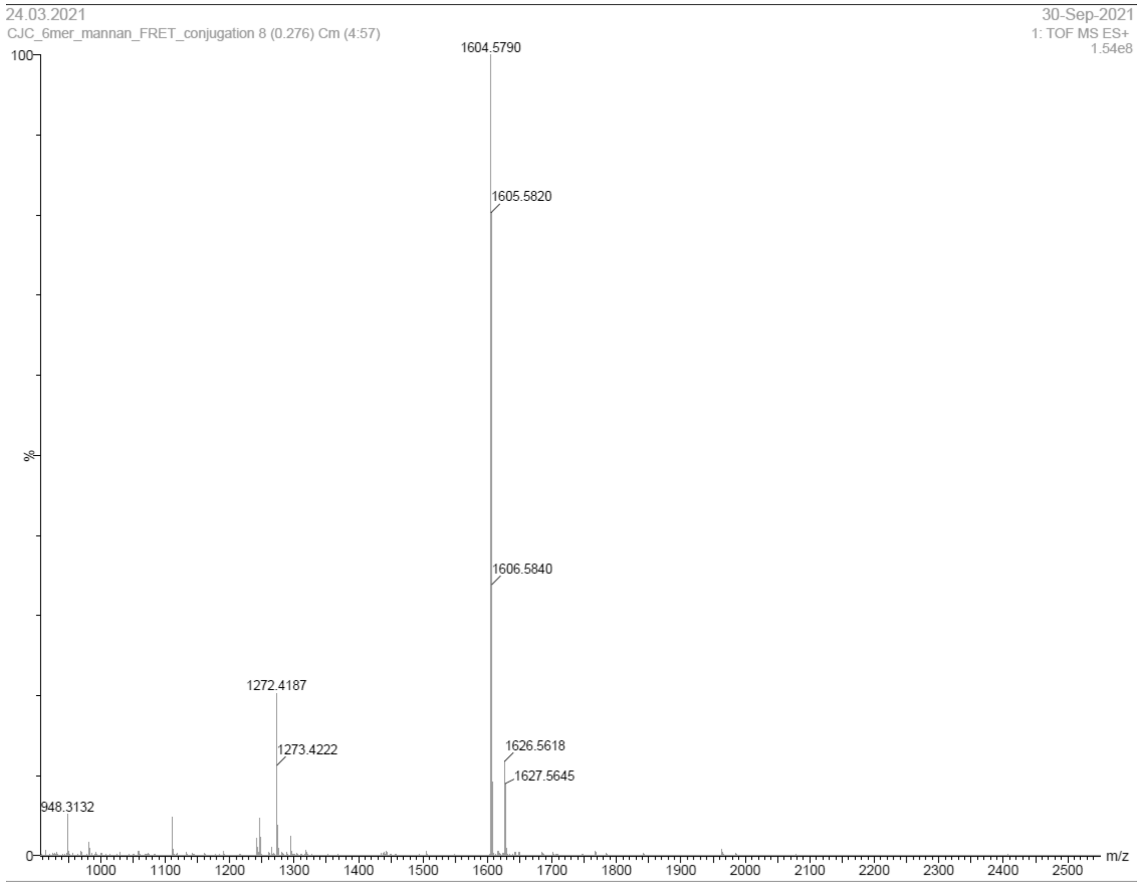

SI Figure 5. Conjugation of fluorescein. a Mannan oligosaccharide after conjugation with fluorescein. **b** Q-TOF of oligosaccharide *m/z* = 1603 $[M+H]^+$.

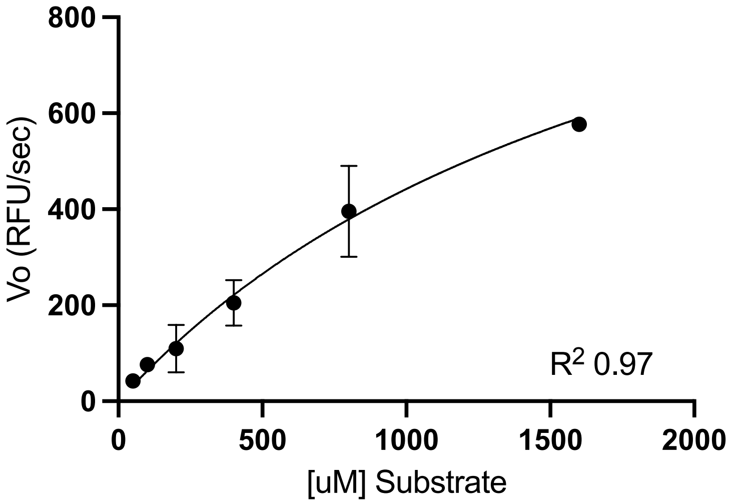

SI Figure 6. Michaelis–Menten kinetics of GH76 mannanase with the α-mannan FRET probe. The reaction was performed using 1 nM purified GH76 enzyme from *Salegentibacter sp.* Hel_I_6 and increasing concentrations of α-mannan FRET probe (50 to 1600 μM). Fluorescence signal corresponding to probe cleavage was recorded, and initial velocities were used to determine kinetic parameters. Error bars represent the standard deviation of triplicate measurements. Fitted parameters: K_m = 2001 μM, V_{max} = 1327 RFU/min, k_{cat} = 1327 s^{-1}, k_{cat}/K_m = 0.663 $\mu M^{-1}s^{-1}$.

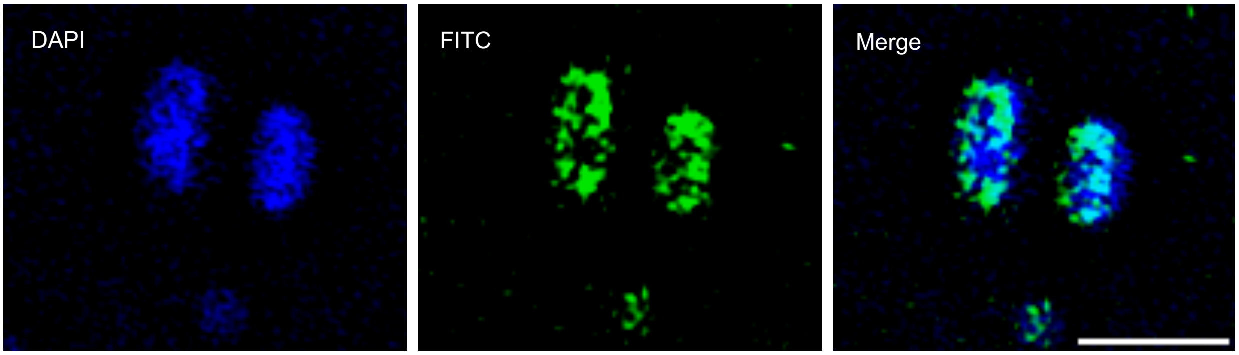

SI Figure 7. STED microscopy of *Salengentibacter sp.* degrading α-mannan FRET probe. Green (FITC) corresponds to the mannan probe signal, and blue (DAPI) labels the cell nuclei. Merge is overlay of FITC and DAPI signals. Scale bar = 2 μM.

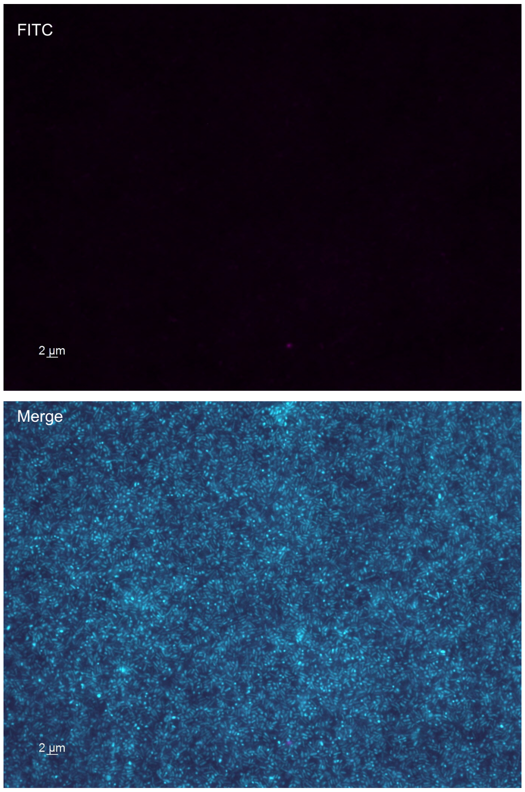

SI Figure 8. Representative microscopy of *F. agariphila* when incubated with the α-mannan FRET probe. Magenta (FITC) corresponds to the mannan probe signal, and blue (DAPI) labels the cell nuclei. Merge is overlay of FITC and DAPI signals. Scale bar = 2 µM.

SI Table 1. **Quantification of α-mannan degradation in microbial communities. ***

Time	***Non-specific carbon*** *(0.5 g/L Baker's yeast)*			***Specific carbon*** *(2 g/L α-mannan)*		
	Total cells (DAPI/mL)	Positive cells (per mL)	Positive (%)	Total cells (DAPI/mL)	Positive cells (per mL)	Positive (%)
15 min	1.7×10^{7}	0	0%	1.01×10^{6}	3.06×10^{2}	0.030%
30 min	1.67×10^{7}	7.20×10^{2}	0.004%	4.78×10^{6}	0	0.0%
90 min	1.18×10^{7}	4.58×10^{2}	0.004%	1.34×10^{6}	0	0.0%
3 h	3.20×10^{7}	1.57×10^{4}	0.049%	1.22×10^{6}	0	0.0%
7 h	3.90×10^{7}	5.91×10^{4}	0.152%	1.06×10^{6}	0	0.0%

* Heat killed control samples from non-specific carbon and specific carbon showed no positive cells.

Automated glycan assembly

Solvents were taken from an anhydrous solvent system (JC Meyer-solvent systems) to prepare activator, acid wash (TMSOTf), and capping solutions. The building blocks were co-evaporated once with toluene and dried under a high vacuum before use. All solutions were freshly prepared and kept under argon during the automation run. Final yields were calculated based on the resin loading. Resin loading was determined by performing a double glycosylation followed by DBU-promoted Fmoc-cleavage and determination of dibenzofulvene formation by measuring its UV absorbance.

Preparation of reagent solutions

Building block solution: Building block (0.09 mmol) was dissolved in CH_2Cl_2 (1 mL).

NIS/TfOH activator solution: Recrystallized NIS (1.56 g) was dissolved in 40 mL of a 2:1 v/v mixture of anhydrous CH_2Cl_2 and anhydrous dioxane. Then, triflic acid (55µL, 0.6 mmol) was added. The solution was kept on an ice bath for the duration of the automation run.

Fmoc deprotection solution: a solution of 20% piperidine in DMF (v/v).

Acid wash solution: TMSOTf (0.45 mL, 2.49 mmol) was dissolved in CH_2Cl_2 (40 mL).

Capping solution: A 50 mL solution of 10% acetic anhydride and 2% methanesulfonic acid in CH_2Cl_2 (v/v) was prepared.

Modules for Automated Solid-Phase Synthesis

Resin preparation for synthesis

40mg (loading of 0.4 mmol/g) of resin was placed in the reaction vessel and swollen in CH_2Cl_2 for 20 min at room temperature prior to synthesis. During this time, all reagent lines needed for the synthesis were washed and primed. Before the first glycosylation, the resin was washed with the DMF, THF, and CH_2Cl_2 (three times each with 2 mL for 25 s).

TMSOTf acidic wash solution (Module a): The resin was swollen in CH_2Cl_2 (2 mL) and the temperature of the reaction vessel was adjusted to -20 °C. Upon reaching the low temperature, TMSOTf solution (1 mL, 0.06 mmol) was added dropwise to the reaction vessel. After bubbling for 3 min, the acidic solution was drained and the resin was washed with CH_2Cl_2 (2 mL) for 25 s.

Action	Cycles	Solution	Amount	T (°C)	Incubation time
Cooling	-	-	-	-20	-
Deliver	1	CH_2Cl_2	2 mL	-20	-
Deliver	1	TMSOTf solution	1 mL	-20	3 min
Wash	1	CH_2Cl_2	1 mL	-20	25 s

Thioglycoside glycosylation (Module b): The building block solution (0.1 mmol of BB in 1 mL of CH_2Cl_2 per glycosylation) was delivered to the reaction vessel. After the set temperature was reached, the reaction was started by dropwise addition of the activator solution (1.0 mL, excess). After completion of the reaction, the solution is drained and the resin was washed with CH_2Cl_2, CH_2Cl_2/dioxane (1:2, v/v, 2 mL for 20 s), and CH_2Cl_2 (twice, each with 2 mL for 25 s). The temperature of the reaction vessel is increased to 25°C for the next module.

Action	Cycles	Solution	Amount	T (°C)	Incubation time
Cooling	-	-	-	-20	-
Deliver	1	BB solution	1 mL	-20	-
Deliver	1	activator solution	1 mL	-20	-
Reaction time	1			-20	15 min
				to 0	35 min
Wash	1	CH_2Cl_2	2 mL	0	25 sec
Wash	1	CH_2Cl_2:Dioxane	2 mL	0	20 sec
Heating	-	-	-	25	-
Wash	1	CH_2Cl_2	2 mL	>0	25 sec

Capping (Module c): The resin was washed twice with DMF (2 mL, 25 s) and the temperature of the reaction vessel was adjusted to 25 °C. Pyridine solution (2 mL, 10% in DMF) was delivered into the reaction vessel. After 1 min, the reaction solution was drained and the resin was washed with CH_2Cl_2 (three times with 3 mL for 25 s). Capping solution (4 mL) was delivered into the reaction vessel. After 20 min, the reaction solution was drained and the resin was washed with CH_2Cl_2 (three times with 3 mL for 25 s).

Action	Cycles	Solution	Amount	T (°C)	Incubation time
Heating	-	-	-	25	-
Wash	2	DMF	2 mL	25	25 s
Deliver	1	10% Py./DMF	2 mL	25	1 min
Wash	3	CH_2Cl_2	2 mL	25	25 s
Deliver	1	Capping solution	4 mL	25	20 min
Wash	3	CH_2Cl_2	2 mL	-20	25 s

Fmoc deprotection with piperidine (**Module d):** The resin was washed with DMF (three times with 2 mL for 25 s) and the temperature of the reaction vessel was adjusted to 25 °C. 2 mL of Fmoc deprotection solution was delivered to the reaction vessel and kept under Ar bubbling. After 5 min, the reaction solution was drained and the resin was washed with DMF (three times with 2 mL for 25 s) and CH_2Cl_2 (five times each with 2 mL for 25 s). The temperature of the reaction vessel was decreased to -20 °C for the next module.

Action	Cycles	Solution	Amount	T (°C)	Incubation time

Wash	3	DMF	2 mL	25	25 s
Deliver	1	Fmoc depr.	2 mL	25	5 min
Wash	3	DMF	2 mL	25	25 s
Wash	5	CH_2Cl_2	2 mL	25	25 s
Cooling	1	-	-	-20	-

Solid-phase synthesis

Methanolysis (Module e): The resin was suspended in anhydrous THF (4.8 mL). Then, 0.2 mL of a solution of NaOMe in MeOH (0.5 M) was added and the resin was shaken at room temperature for 16 h. The resin was then washed successively with THF, CH_2Cl_2, methanol, and CH_2Cl_2.

Photocleavage from the solid support (Module f): Glycans were cleaved from the solid support using a batch-flow photoreactor. The resin-bound glycan (~40 mg) was suspended in DMF (4 mL) under the irradiation of an LED lamp (370nm), with stirring for 24 hours. The solution was separated from the resin using a fritted syringe and concentrated under vacuum.

Peptide coupling conditions (Module g) Resin was swollen for 20 min in CH_2Cl_2 and then the Fmoc protecting group was removed by treatment with a solution of 20% piperidine in DMF (2×10 min). The resin was then washed with CH_2Cl_2 (3×1 min) and DMF (2×1 min). PyBOP (8 eq) and boc-β-alanine (8 eq.) were dissolved in DMF in a glass vial, then N-Methylmorpholine (16 equiv.) was added. The mixture was left for 4 min and then added to the resin, and left at room temperature for 90 min. The resin is washed with DCM (3×1 min) and DMF (2×1 min).

Solution-phase synthesis

Hydrogenolysis (Module h): The crude compound was dissolved in 4 mL of THF: *t*-BuOH: H_2O (60:10:30). 5% Pd-C (200 mg) was added and the reaction was stirred under H_2 atmosphere for 12 h. The reaction was filtered through a pad of celite and washed with *t*-BuOH and H_2O. The filtrates were concentrated *in vacuum*, and dissolved in 3.0 mL of water for RP-HPLC purification.

NHS coupling of fluorescein or rhodamine (Module i) The glycan was dissolved in anhydrous DMSO and NHS-activated dye was added (3 eq). The reaction was tracked via Q-TOF. If the conjugation was incomplete after 5 hours, further equivalents of NHS-dye were added.

HPLC analysis and purification

Analytical traces of crude and pure compounds were collected using an analytic HPLC Agilent 1200 Series (**Methods 1** and **2**) and preparative RP-HPLC Agilent 1200 Series (**Method 3**).

SI Method 1, analytic NP-HPLC: (YMC-Diol-300 column, 150 x 4.6 mm) flow rate of 1.0 mL / min with Hex – 20% EtOAc as eluent [isocratic 20% EtOAc (5 min), linear gradient to 55% EtOAc (35 min), linear gradient to 100% EtOAc (5 min)].

SI Method 2 analytic RP-HPLC: (Phenomenex, luna C5 column, 250 x 4.6 mm, 5 µm), flow rate of 1.0 mL /min with ACN/H_2O (0.1% formic acid) as eluents [isocratic 5% ACN (5 min), linear gradient to 100% ACN (30 min)].

SI Method 3 prep RP-HPLC: (Phenomenex, luna C5 column, 250 x 4.6 mm, 5 µm), flow rate of 3.0 mL /min with ACN/H_2O (0.1% formic acid) as eluents [isocratic 5% ACN (5 min), linear gradient to 100% ACN (30 min)].

Following purification, all products were lyophilized on a Christ Alpha 2-4 LD plus freeze dryer before characterization.

Compound characterization

Compound 1 Mannan FRET probe

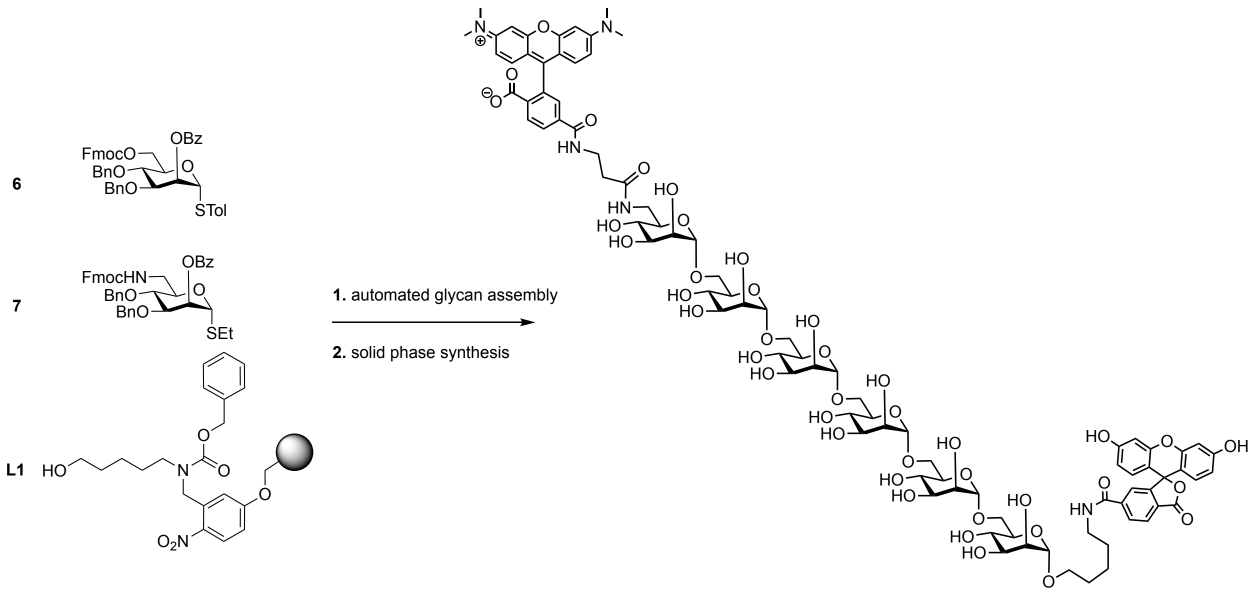

		Modules	Notes
**1. AGA**			L1
	**1**	a, b, c, d	x5
	**2**	a, b, c, d	x1
	**Step**	**Module**	**Notes**
**2. Solid-phase synthesis**	peptide coupling	i	Boc-β-alanine
	methanolysis	g	
	photocleavage	i	
	**Step**	**Module**	**Notes**
**3. Solution-phase**	hydrogenolysis	j1	
	purification	method 3	

The desired fractions were then collected and lyophilized to yield 0.9 mg (4.5%). **^{1}H NMR** (600 MHz, D_2O) δ 8.01 – 7.99 (m, 1H, $H_{ar\ rhodamine}$), 7.92 (dd, *J* = 8.0, 5.3 Hz, 2H, $H_{ar\ fluorescein}$), 7.86 (d, *J* = 8.1 Hz, 1H, $H_{ar\ rhodamine}$), 7.55 (s, 1H, $H_{ar\ rhodamine}$), 7.47 (s, 1H, $H_{ar\ rhodamine}$), 7.25 (t, *J* = 8.8 Hz, 1H, $H_{ar\ rhodamine}$), 7.08 (t, *J* = 8.5 Hz, 2H, $H_{ar\ fluorescein}$), 6.93 – 6.90 (m, 3H, $H_{ar\ rhodamine}$), 6.83 (t, *J* = 9.4 Hz, 2H, $H_{ar\ rhodamine}$), 6.58 (d, *J* = 8.4 Hz, 2H, $H_{ar\ rhodamine}$), 6.37 – 6.33 (m, 4H, $H_{ar\ fluorescein}$), 4.89 (s, 1H, H-1), 4.87 (s, 1H, H-1), 4.85 (s, 2H, H-1), 4.02 – 3.93 (m, 8H), 3.94 – 3.76 (m, 9H), 3.76 – 3.63 (m, 4H, $CH_{2\ alanine\ linker}$), 3.62 – 3.51 (m, 3H), 3.36 (t, *J* = 7.0 Hz, 2H, $CH_{2\ alanine\ linker}$), 3.17 (d, *J* = 8.2 Hz, 12H, $CH_{3\ rhodamine}$), 2.69 – 2.62 (m, 3H, $CH_{2\ alanine\ linker}$), 1.68 – 1.61 (m, 7H, $CH_{2\ amino\ pentyl\ linker}$), 1.52 – 1.42 (m, 2H, $CH_{2\ amino\ pentyl\ linker}$). **^{13}C NMR‡** (151 MHz, D2O) δ 131.2 ($H_{ar\ rhodamine}$), 131.0 ($H_{ar\ rhodamine}$), 130.8 ($H_{ar\ fluorescein}$), 129.1 ($H_{ar\ rhodamine}$), 128.9 ($H_{ar\ fluorescein}$), 128.7 ($H_{ar\ fluorescein}$), 128.7 ($H_{ar\ rhodamine}$), 128.7, 122.8 ($H_{ar\ fluorescein}$), 114.1 ($H_{ar\ rhodamine}$), 113.9 ($H_{ar\ rhodamine}$), 103.8 ($H_{ar\ fluorescein}$), 99.9 (H-1_{man}), 99.4 (H-1_{man}), 99.4 (H-1_{man}), 99.2 (H-1_{man}), 96.3, 96.0, 70.9, 70.9, 70.2, 70.1, 69.7 ($_{amino\ pentyl\ linker}$), 66.9, 65.7 (H-6_{man}), 40.1 ($CH_{2\ amino\ pentyl\ linker}$), 40.1 ($CH_{3\ rhodamine}$), 36.7 ($CH_{2\ alanine\ linker}$), 35.3 ($CH_{2\ alanine\ linker}$), 28.3 ($CH_{2\ amino\ pentyl\ linker}$), 23.0 ($CH_{2\ amino\ pentyl\ linker}$). **HRMS** Q-TOF MS calcd. $C_{90}H_{111}N_5O_{41}$ for $[M+H]^+$ 1917.6749, found 1917.6504.

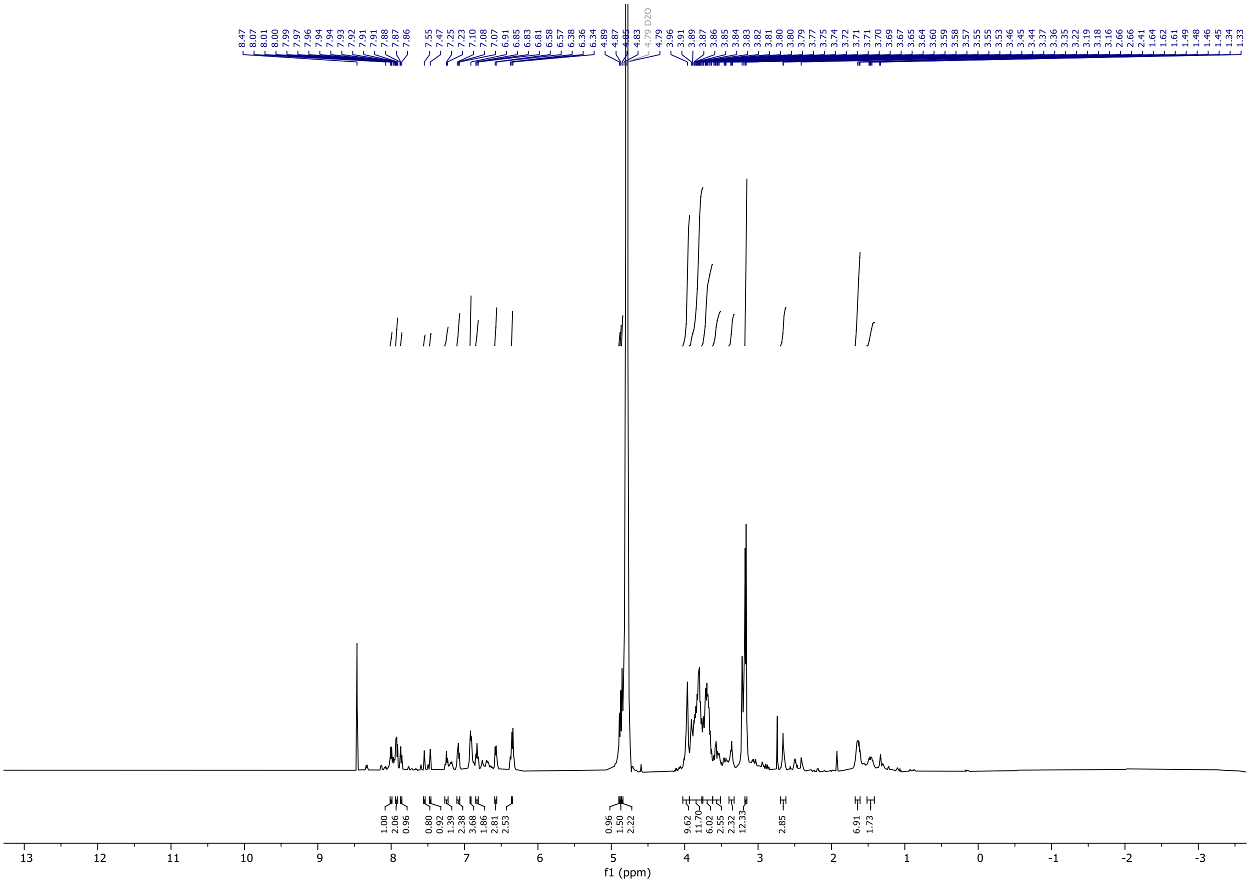

^{1}H NMR

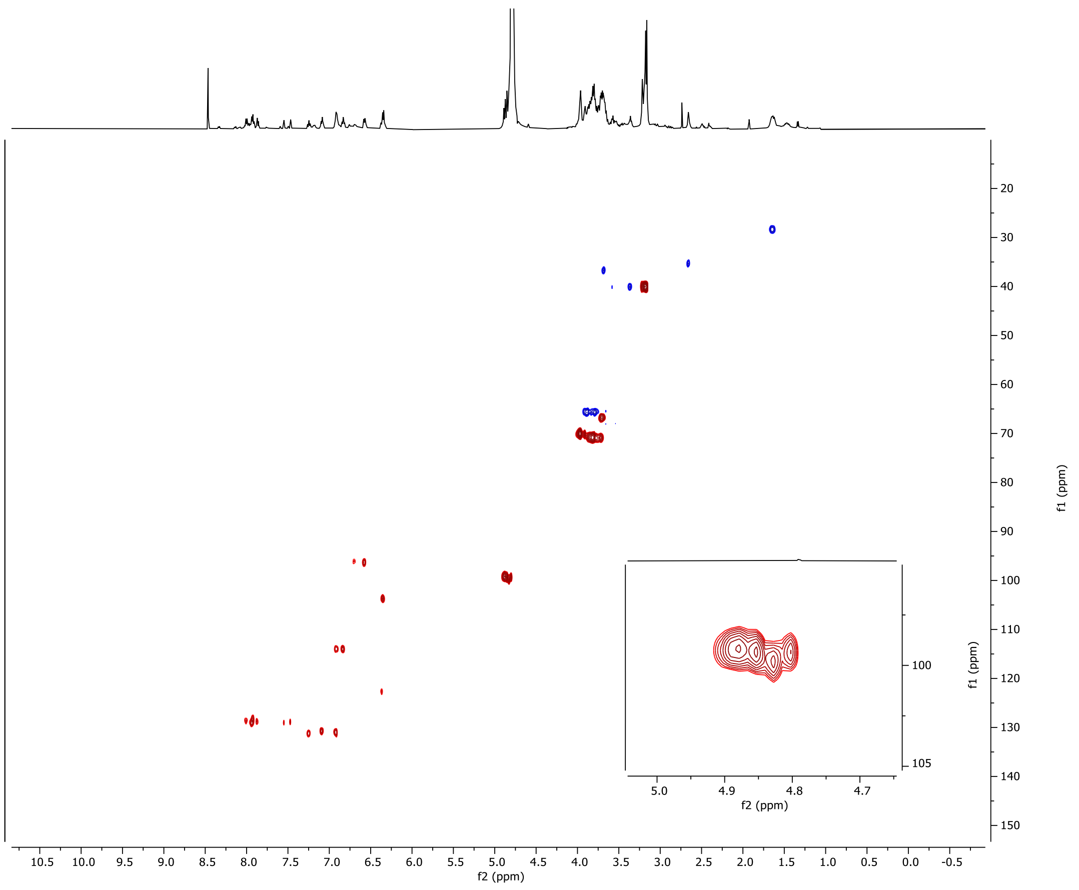

^{1}H-^{13}C HSQC NMR

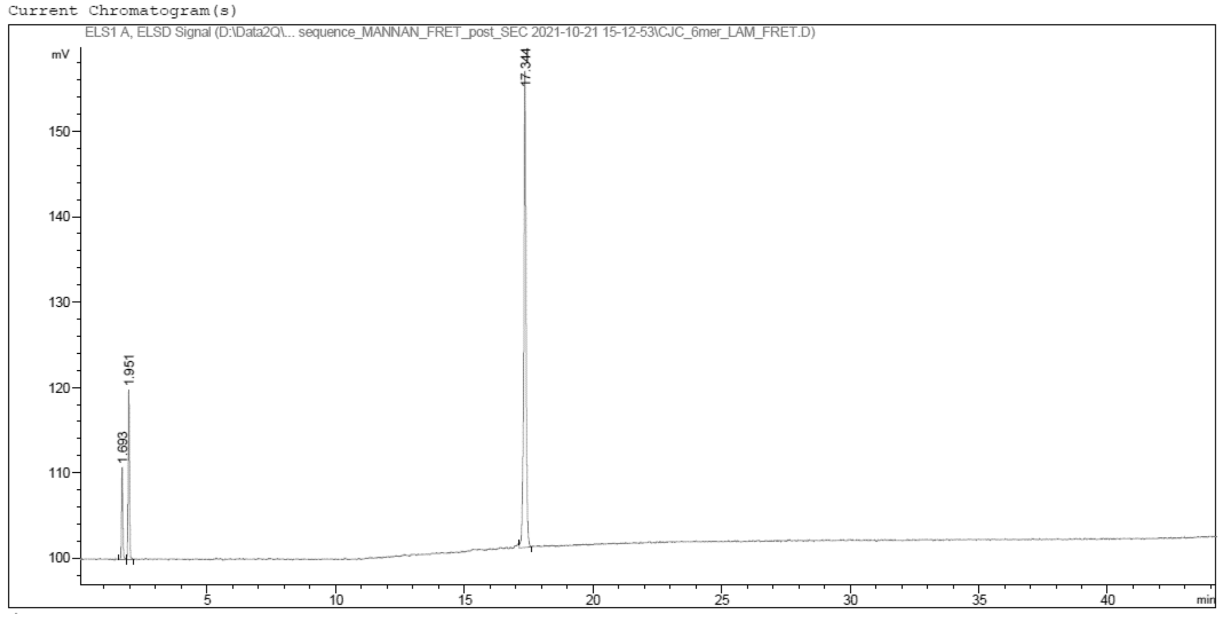

RP-HPLC (C5 Luna, ELSD trace) mannan FRET probe.

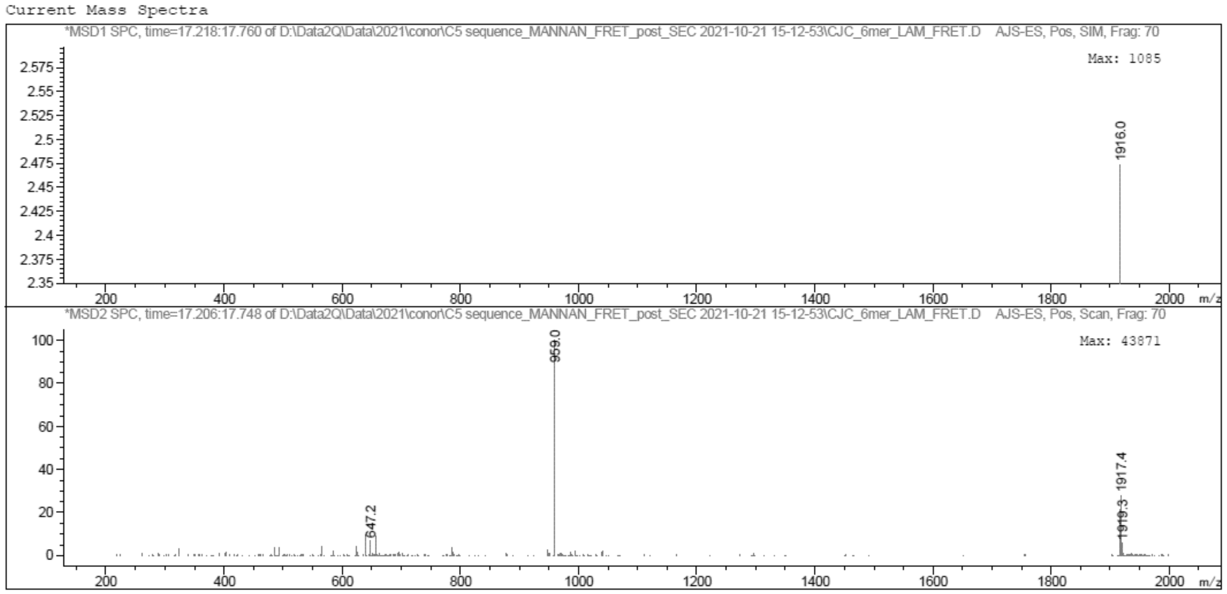

MSD spectra of peak at 17 mins from RP-HPLC (C5 Luna, ELSD trace) mannan

Overexpression and purification of GH76

Protein expression of GH76 followed published protcols.[3] Briefly, *Escherichia coli* BL21DE3 cells harbouring pET28a-GH76 were cultured in 1 L lysogeny broth (LB) medium supplemented with 50 µg/mL kanamycin at 37 °C until the mid-exponential phase (OD_{600nm} 0.6–0.8). Recombinant gene expression was induced by addition of 0.3 mM (final concentration) isopropyl β-D-1-thiogalactopyranoside (IPTG) and further incubation at 16 °C for ~16 h.

In case of the ShGH76 clones, cells were harvested by centrifugation and stored at −20 °C. Cell lysis was conducted chemically. Frozen cell pellets were resuspended in 20 mL sucrose solution (25% w/v sucrose, 50 mM Tris HCl, pH 8.0). Lysozyme was added at a concentration of 1 mg/mL and the cells were subsequently incubated for 10 min. at room temperature with stirring 40 mL of deoxycholate solution (1% w/v deoxycholate, 1% w/v Triton X-100, and 100 mM NaCl) was added followed by $MgCl_2$ to a final concentration of 1 mM and DNase to a concentration of 1 mg/mL. The resulting lysate was centrifuged at 16,000 × *g* for 45 min at 4 °C. For purification, clarified lysate was applied to a 5 mL prepacked IMAC column (GE Healthcare Life Sciences, Marlborough, MA, USA) equilibrated in buffer A (20 mM Tris-HCl, pH 8 and 500 mM NaCl) using an ÄKTA start FPLC (fast protein liquid chromatography) system (Cytiva, Marlborough, MA, USA). The column was washed with buffer A and the His-tagged protein was eluted using a gradient of imidazole to 500 mM in Buffer A. The purified protein was concentrated using a stirred cell ultrafiltration device with a 10 kDa membrane and subsequently further purified using size exclusion chromatography [using HiPrep Sephacryl S200 HR column (Cytiva, Marlborough, MA, USA)] in 20 mM Tris-HCl, pH 8 with 250 mM NaCl. Finally, the protein was concentrated to 20 mg/mL prior to further experiments as determined by absorbance at 280 nm using the extinction coefficient of 2.083 for ShGH76.

Bacteria culture

Bacteria Culture of *Gramella forsetii*, *Saligentibacter* Hel1_6, *Formosa agariphila* and *Formosa* Hel1_33_131 (alias Formosa B) were cultured in HaHa100V medium following published protocols.[4] Upon reaching mid-log phase, cultures were diluted 1:100 (v/v) into HaHa3V, a low-carbon medium, to initiate induction. To induce GH76 expression, yeast α-mannan was added at a final concentration of 100 mg/L to all cultures. Cultures were incubated at 20 °C with gentle agitation.

FRET mannan uptake studies with marine bacteria

Cultures of marine bacteria grown in HaHa100V medium following published protocols.[4] Cells were harvested at mid- to late-exponential growth phase (OD_{600nm} 0.6–1.0), pelleted by centrifugation (4700 × g for 5 min), and washed three times with a defined minimal medium composed of: NH_4SO_4 (8.5 mM), Na_2CO_3 (9.4 mM), l-cysteine-free base (4.1 mM), KH_2PO_4 pH 7.2 (100 mM), $FESO_4 \cdot 7H_2O$ (1.4 μM), vitamin K_3 (1 μg mL^{-1}), vitamin B_{12} (5 ng mL^{-1}), NaCl (15.4 mM), $CaCl_2$ (0.24 mM), $MgCl_2 \cdot 6H_2O$ (98 μM), $MnCl_2 \cdot 4H_2O$ (50 μM), $CoCl_2 \cdot 6H_2O$ (42 μM), resazurin (1 μg mL^{-1}), and histidine/hematin solution (2 μL mL^{-1} v/v; 1.9 μM hematin/200 μM l-histidine; 1000× stock solution).

Microscopy samples: Following the washes, cells were resuspended in 1 mL phosphate-buffered saline (PBS, pH 7.4), and 2.5 μM of the mannan FRET probe was added. Samples were incubated for 60 min at room temperature. After incubation, cells were pelleted by centrifugation, washed three times with PBS, and fixed in 1% formaldehyde (diluted from stock in PBS). Fixed cells were stored at 4 °C overnight before imaging.

Plate Reader Assays (Cell Lysates): For lysate experiments, cells were pelleted (5000 × g, 20 min) and lysed using BugBuster® reagent (Merck) according to the manufacturer's instructions. Lysates were resuspended in PBS (pH 7.4), and 1 μM of the mannan FRET probe was added. After 60 min of incubation at room temperature, 50 μL aliquots were transferred into black 96-well microplates. Fluorescence was measured using a microplate reader with excitation at 495 nm and emission detection at 520 nm.

Epifluorescence microscopy

Epifluorescence microscopy was performed as previously described.[5] Briefly, fixed bacterial cells were filtered onto 25 mm polycarbonate filters (0.2 μm pore size, Millipore) using a gentle vacuum (<200 mbar). Cells were counterstained with DAPI and mounted using a 4:1 mixture of Citifluor (Electron Microscopy Sciences, USA) and VECTASHIELD (Vector Laboratories, Germany). Imaging was carried out using a Zeiss Axioskop 2 mot plus fluorescence microscope equipped with Axiovision software (Zeiss, Germany).

Microbial community culture

Seawater was collected from a marine aquarium maintained at the Max Planck Institute for Marine Microbiology and processed following established protocols.[4] Two cultures were prepared: one supplemented with baker's yeast (0.5 g/L) and the other with α-mannan (2 g/L), and incubated for three days at 20 °C. Subsequently, each culture was treated with 5 μM of the mannan FRET probe and sampled at five time points ranging from 15 minutes to 7 hours. At each time point, samples were fixed by adding 37% (v/v) formaldehyde to a final concentration of 1% (v/v) and stored at room temperature until analysis.

Automated fluorescence microscopy

Automated fluorescence microscopy was complete as described previously.[5,6] For all the time points, the cells were 0.2 μM filtered and counter-stained with 4′,6-diamidino-2-phenylindole (DAPI) and subsequently mounted using a Citifluor/VectaShield (4:1) mounting solution. Substrate-stained cells were visualised and enumerated using a fully automated microscope imaging system, on a Zeiss AxioImager.Z2 microscope stand (Carl Zeiss MicroImaging GmbH, Göttingen, Germany) with a cooled charged-coupled-device (CCD) camera (AxioCam MRm; Carl Zeiss) and a Colibri LED light source (Carl Zeiss) with three light-emitting diodes (UV-emitting LED, 365±4.5 nm for DAPI; blue emitting LED, 470±14 nm for FLA-PS 488; red-emitting LED, 590±17.5 nm for the tyramide Alexa 594), combined with the HE-62 multifilter module (Carl Zeiss). This

module consists of a triple emission filter TBP 425 (±25), 527 (±27), LP 615, including a triple beam splitter of TFT 395/495/610.

All automatic cell counts were validated using manual cell counting. Briefly automated cell counting was carried out by initially acquiring images (using a 63 × magnification and 1.4 numerical aperture oil emersion plan apochromatic objective (Carl Zeiss), at selected wavelengths (DAPI, FRET mannan), of a previously defined set of coordinates consisting of a minimum of 46 fields of view on each sample filter. Subsequently, the images were imported into the ACMETOOL2.0 (http://www.technobiology.ch/index.php?id=acmetool) image analysis software. From the images, cells were deemed 'substrate stained' if they showed a positive signal in both the DAPI and FRET mannan images. Additionally, these signals had to have a minimum overlap of 30%, a minimum area of 17 or 30 pixel (0.17–0.3 μm^2) (DAPI signal and FRET mannan, respectively) and a minimum signal background ratio of 1 or 2.5 (DAPI and FRET mannan signals, respectively).

Super resolution STED microscopy

STED microscopy was performed as described previously.[6] For sample preparation, 30 µL of fixed cell culture was heat fixed at 40°C to poly-D-lysine-coated coverslips (#1.5, thickness 0.17mm). Residual salts from the medium were removed by gently washing the coverslips in Milli-Q water. For both stimulated emission depletion (STED) cells were mounted using ProLong™ Diamond Antifade Mountant (Thermo Fisher Scientific, Germany).

FRET mannan uptake was visualized using an Abberior Instruments STED microscope (Abberior Instruments GmbH, Germany). Excitation was performed with a 485 nm laser, and emission was collected using a 525/50 nm bandpass filter. STED depletion was achieved with a 595 nm laser. Imaging settings included a 70 µm pinhole, 20 nm pixel size, 20 µs dwell time, and nine-line accumulation to enhance signal-to-noise ratio.

References

(1) Ricardo, M. G.; Seeberger, P. H.; Ricardo, M. G.; Seeberger, P. H. Merging Solid-Phase Peptide Synthesis and Automated Glycan Assembly to Prepare Lipid-Peptide-Glycan Chimeras. *Chem. Eur. J.* **2023**, e202301678. https://doi.org/10.1002/CHEM.202301678.

(2) Weishaupt, M. W.; Hahm, H. S.; Geissner, A.; Seeberger, P. H. Automated Glycan Assembly of Branched β-(1,3)-Glucans to Identify Antibody Epitopes. *Chem. Comm.* **2017**, *53* (25), 3591–3594. https://doi.org/10.1039/c7cc00520b.

(3) Solanki, V.; Krüger, K.; Crawford, C. J.; Pardo-Vargas, A.; Danglad-Flores, J.; Hoang, K. L. M.; Klassen, L.; Abbott, D. W.; Seeberger, P. H.; Amann, R. I.; Teeling, H.; Hehemann, J.-H. Glycoside Hydrolase from the GH76 Family Indicates That Marine Salegentibacter Sp. Hel_I_6 Consumes Alpha-Mannan from Fungi. *ISME J.* **2022**, *16* (7), 1818–1830. https://doi.org/10.1038/s41396-022-01223-w.

(4) Hahnke, R. L.; Bennke, C. M.; Fuchs, B. M.; Mann, A. J.; Rhiel, E.; Teeling, H.; Amann, R.; Harder, J. Dilution Cultivation of Marine Heterotrophic Bacteria Abundant after a Spring Phytoplankton Bloom in the North Sea. *Environ. Microbiol.* **2015**, *17* (10), 3515–3526. https://doi.org/10.1111/1462-2920.12479,.

(5) Hehemann, J. H.; Reintjes, G.; Klassen, L.; Smith, A. D.; Ndeh, D.; Arnosti, C.; Amann, R.; Abbott, D. W. Single Cell Fluorescence Imaging of Glycan Uptake by Intestinal Bacteria. *ISME J.* **2019**, *13* (7), 1883–1889. https://doi.org/10.1038/S41396-019-0406-Z.

(6) Reintjes, G.; Arnosti, C.; Fuchs, B. M.; Amann, R. An Alternative Polysaccharide Uptake Mechanism of Marine Bacteria. *ISME J.* **2017**, *11* (7), 1640–1650. https://doi.org/10.1038/ismej.2017.26.
